# Objective Monitoring of Facioscapulohumeral Dystrophy During Clinical Trials Using a Smartphone App and Wearables: Observational Study

**DOI:** 10.2196/31775

**Published:** 2022-09-13

**Authors:** Ghobad Maleki, Ahnjili Zhuparris, Ingrid Koopmans, Robert J Doll, Nicoline Voet, Adam Cohen, Emilie van Brummelen, Geert Jan Groeneveld, Joris De Maeyer

**Affiliations:** 1 Centre for Human Drug Research Leiden Netherlands; 2 Leiden University Medical Center Leiden Netherlands; 3 Radboud University Medical Center Nijmegen Netherlands; 4 Klimmendaal Arnhem Netherlands; 5 Facio Therapies Leiden Netherlands

**Keywords:** facioscapulohumeral dystrophy, FSHD, smartphone, wearables, machine learning, classification, mobile phone

## Abstract

**Background:**

Facioscapulohumeral dystrophy (FSHD) is a progressive muscle dystrophy disorder leading to significant disability. Currently, FSHD symptom severity is assessed by clinical assessments such as the FSHD clinical score and the Timed Up-and-Go test. These assessments are limited in their ability to capture changes continuously and the full impact of the disease on patients’ quality of life. Real-world data related to physical activity, sleep, and social behavior could potentially provide additional insight into the impact of the disease and might be useful in assessing treatment effects on aspects that are important contributors to the functioning and well-being of patients with FSHD.

**Objective:**

This study investigated the feasibility of using smartphones and wearables to capture symptoms related to FSHD based on a continuous collection of multiple features, such as the number of steps, sleep, and app use. We also identified features that can be used to differentiate between patients with FSHD and non-FSHD controls.

**Methods:**

In this exploratory noninterventional study, 58 participants (n=38, 66%, patients with FSHD and n=20, 34%, non-FSHD controls) were monitored using a smartphone monitoring app for 6 weeks. On the first and last day of the study period, clinicians assessed the participants’ FSHD clinical score and Timed Up-and-Go test time. Participants installed the app on their Android smartphones, were given a smartwatch, and were instructed to measure their weight and blood pressure on a weekly basis using a scale and blood pressure monitor. The user experience and perceived burden of the app on participants’ smartphones were assessed at 6 weeks using a questionnaire. With the data collected, we sought to identify the behavioral features that were most salient in distinguishing the 2 groups (patients with FSHD and non-FSHD controls) and the optimal time window to perform the classification.

**Results:**

Overall, the participants stated that the app was well tolerated, but 67% (39/58) noticed a difference in battery life using all 6 weeks of data, we classified patients with FSHD and non-FSHD controls with 93% accuracy, 100% sensitivity, and 80% specificity. We found that the optimal time window for the classification is the first day of data collection and the first week of data collection, which yielded an accuracy, sensitivity, and specificity of 95.8%, 100%, and 94.4%, respectively. Features relating to smartphone acceleration, app use, location, physical activity, sleep, and call behavior were the most salient features for the classification.

**Conclusions:**

Remotely monitored data collection allowed for the collection of daily activity data in patients with FSHD and non-FSHD controls for 6 weeks. We demonstrated the initial ability to detect differences in features in patients with FSHD and non-FSHD controls using smartphones and wearables, mainly based on data related to physical and social activity.

**Trial Registration:**

ClinicalTrials.gov NCT04999735; https://www.clinicaltrials.gov/ct2/show/NCT04999735

## Introduction

### Background

A recent Dutch population study on facioscapulohumeral dystrophy (FSHD) estimated that approximately 2000 people in the Netherlands and approximately 800,000 people worldwide are living with FSHD [1]. Often, early symptoms include difficulty whistling, smiling, and closing the eyelids while asleep. Weakening of the facial muscles is generally followed by scapular winging. This abnormal positioning of the shoulder bone impairs the movement of the shoulders and arms. Further weakening of the muscles is commonly observed in the upper arms and may progress to the hip girdle and lower legs in severe cases. Less visible symptoms of FSHD are chronic pain and fatigue [2]. In addition to the physical symptoms the diagnosis of FSHD comes with an emotional and social burden. The highly variable and unpredictable progression of the disease can have a strong impact on the quality of life [3,4]: 90% of the affected individuals have visible symptoms by the age of 20 years and 1 in 5 patients with FSHD eventually becomes wheelchair dependent [5].

No therapy is currently available that stops the progression of FSHD [6-9]. Patients thus have to rely on symptomatic treatment such as medical devices or surgical intervention [2]. The development of novel treatment options to delay or halt disease progression is currently under investigation. However, measuring the effect of such new treatments is complicated because disease progression is slow and no objective surrogate end points, predictive for clinical benefit, have been established. App-based technologies may help to more closely monitor FSHD symptom progression and evaluate potential treatment effects on a continuous basis.

Currently, FSHD symptom severity is assessed by clinical scoring of symptoms such as the FSHD clinical score or mobility performance tests such as the Timed Up-and-Go test (TUG) and Reachable Workspace assessment [10-12]. These clinical severity and functional scores have several drawbacks. Scores change very slowly over time [13], are assessed in a clinic at 1 specific moment, and do not cover the implications of the disease on social and physical activity during daily life. The progressive muscle weakness characterizing FSHD leads to massive changes in the way people live their lives, affecting how they get around, how they complete daily activities, and whether they can work or care for children. Therefore, assessing disease severity may be improved by not only measuring muscle function but also evaluating social and physical activity data. This study aimed to address this by first classifying disease using a smartphone app and wearables to continuously remotely monitor features relating to biometric, physical, and social activities of patients with FSHD in comparison with those of non-FSHD controls. Subsequently, we performed a second analysis in which we aimed to assess disease severity. This analysis will be described in a different paper.

### Objectives

We investigated the feasibility of remotely monitoring multiple features such as step count, sleep, app use, and location tracking in patients with FSHD and non-FSHD controls. First, we evaluated the participants’ tolerability of these devices. We then characterized the patients with FSHD and non-FSHD controls in terms of composites of social, physical, and biometric activities. We sought to (1) distinguish patients with FSHD from non-FSHD controls using a classification machine learning model and determine the minimum monitoring window needed to perform the classification and (2) identify which of the remotely monitored features were most salient in differentiating between the 2 groups.

## Methods

### Study Overview

We conducted a cross-sectional, noninterventional study in patients with FSHD and non-FSHD controls. A total of 58 participants (n=38, 66%, patients with genetically confirmed FSHD and n=20, 34%, non-FSHD controls) were included in this study at the Centre for Human Drug Research (CHDR) in Leiden, The Netherlands, between April 2019 and October 2019. Patients were recruited from The Netherlands and Belgium.

### Ethics Approval

This study was performed in compliance with International Council for Harmonisation Good Clinical Practice and approved by the Stichting Beoordeling Ethiek Biomedisch Onderzoek Medical Ethics Committee (Assen, The Netherlands; CCMO number NL69288.056.19) according to Wet medisch-wetenschappelijk onderzoek met mensen (Dutch law on medical-scientific research with humans).

### Patient Population

To represent the clinical FSHD spectrum based on symptom severity and age, up to 40 patients with FSHD (and also 20 control participants) were deemed sufficient. As this study was exploratory, sample size was not based on power calculations.

Eligible patients with FSHD were aged >16 years, had genetically confirmed FSHD (FSHD1 or FSHD2), were symptomatic as demonstrated by the FSHD clinical score of >0, and had an Android phone that they used as their main phone or were willing to use one for the duration of the study period. Patients with any comorbidity, expected to affect the measurements, were excluded. Eligible control participants were included using the same inclusion and exclusion criteria that were used to recruit the patients, except they did not have a diagnosis or symptoms of FSHD.

### Data Collection

#### Clinical Assessments

On the first and last days of the study period, the FSHD clinical score assessment was performed in the group consisting of patients with FSHD, whereas the TUG was performed in both groups. On day 42 in both groups the user experience was assessed and the perceived burden questionnaire (Multimedia Appendix 1) administered.

The FSHD clinical score is a standardized clinical score that quantifies muscle weakness by combining the functional evaluations of the 6 muscle groups affected in FSHD. The scale is divided into 6 independent sections that assess the strength and the functionality of facial muscles, scapular girdle muscles, upper limb muscles, distal leg muscles, pelvic girdle muscles, and abdominal muscles [11]. The TUG assesses mobility and balance by measuring the time it takes for a participant to stand up from a seated position in a chair, walk 3 meters, turn around, walk back 3 meters, and sit down again [12]. The user experience and perceived burden questionnaire was developed by the CHDR to measure the impact of remote monitoring of apps on smartphone performance. The questions are based on the overall experience of CHDR with mobile apps.

#### Remote Monitoring Platform

All participants were remotely monitored using the CHDR Monitoring Remotely (CHDR MORE) platform for 42 days. CHDR MORE is a highly customizable platform that allows remote monitoring of participants using smartphones and wearables. The infrastructure used includes an Android app to collect data from smartphone sensors and a connection to the Withings Health (Withings) web-based platform to collect wearable data. All collected features are described in Table 1.

**Table 1:** Overview of all smartphone and wearable sensors used in this study and their respective extracted features.

Device and sensor		Features
**Smartphone**		
	Accelerometer	Maximum magnitude of the acceleration: 98%
	Apps	Number of times an app is opened; amount of time app is open in foreground
	GPS	Total kilometers traveled per day; average kilometers traveled per trip; 95% maximum distance from home
	Google Places	Number of unique places visited; time spent at each unique location
	Calls	Number of outgoing, incoming, and missed calls; number of calls from known and unknown contacts
	Microphone	Percentage of time a human voice is present
**Wearables (Withings)**		
	Watch step count	Total step count; mean steps per minute; mean steps per hour; maximum steps per hour
	Watch heart rate	Heart rate: 5%, 50%, and 95% ranges and SD of heart rate percentage of time spent in resting heart rate
	Watch sleep	Awake as well as light and deep sleep duration (minutes); number of awake as well as light and deep sleep periods; time to fall asleep (minutes)
	Watch physical activity	Soft, moderate, and hard activity duration
	Blood pressure monitor	Systolic and diastolic blood pressure
	Scale	Weight (kg); muscle mass (kg); bone mass (kg); body fat (%); body water (%)

#### Smartwatch, Smart Scale, and Blood Pressure Monitor

In total, three commercially available Withings devices were used: (1) heart rate, step count, and sleep patterns were assessed by the Withings Steel HR smartwatch; (2) weight, BMI, and skeletal muscle mass were assessed by the Withings Body+ scale; and (3) systolic blood pressure and diastolic blood pressure were assessed by the Withings blood pressure monitor. Data from the Withings devices were collected on the phone using Bluetooth and sent to the Withings storage servers before being transferred to a CHDR server. Participants were instructed to wear the Withings Steel HR smartwatch continuously for the duration of the study, and they measured their weight and blood pressure themselves weekly using the Withings Body+ scale and Withings blood pressure monitor, respectively.

#### Privacy

The data collection as part of this study may raise privacy and data safety concerns. Therefore, during development of the CHDR MORE app, we addressed these concerns by building in several measures to maximize privacy for all participants. First, all data sources such as SMS text messaging logs, phone calls, and microphone activation only report summative outcomes. These sources cannot send the content of messages or whole recordings to the CHDR servers. In addition, location data only report relative location instead of absolute GPS coordinates. Furthermore, all calculations such as human voice detection are performed on the Android phone itself and removed afterward and all personal data are coded and safely stored on certified CHDR servers.

### Statistical Analysis

#### Data Preprocessing

The data preprocessing and analysis pipelines were developed using Python (version 3.6.0; Python Software Foundation). The Python library scikit-learn was used for the feature extraction and the development of the machine learning models [14]. All data were manually and visually inspected for missing data and outlier data. The identified outliers (eg, traveling 10,000 kilometers in a single day) were subsequently removed from the analysis. Missing or excluded data points were not imputed.

#### Feature Extraction

As disease progression in FSHD is gradual, the FSHD clinical scores and TUG scores were expected to remain stable during the 6-week period. The daily features were therefore averaged across a defined time window (see the *Identification of Optimal Time Window* section for more information). Table 1 provides a simplified overview of the features that were extracted from the CHDR MORE app and Withings sensors.

#### Feature Selection

Before fitting the classification models to the data set, features were excluded using manual and automated feature selection. The authors (AZ, RJD, AC, EvB, GJG, and JDM) of this paper manually excluded features based on the degree of missing data and the clinical relevance of the feature (eg, time spent on home and house apps were deemed clinically irrelevant). For the automated feature selection, variance inflation factor and stepwise regression were used to exclude multi-collinear features or features that did not provide additive information, respectively.

#### Classification Models

We used 4 categories of data sets for the classification of patients with FSHD and non-FSHD controls. These categories include the composite data (all features), social data (smartphone features relating to social location, social and communication app use, and phone calls), physical activity data (smartwatch features), and biometric data (scale and blood pressure monitor features). We compared the performance of the logistic regression, random forest, and support vector machine classification models (Multimedia Appendix 2 [15-22]). The performance of these classification models was evaluated by the accuracy, sensitivity, specificity, and Matthews correlation coefficient (MCC). A grid search was performed to find the optimal hyperparameters (the parameters that determine the model’s structure) that would yield the highest sensitivity and specificity for each model. Furthermore, we performed a 5-fold stratified cross-validation. Cross-validation is a resampling method used to evaluate the prediction performance of the classification models. The data were divided into 5 equal subsets, with the same FSHD-to–non-FSHD ratio within each subset; the model was trained on 4 (80%) partitions of the data and tested on 1 (20%) partition. This procedure was repeated 5 times, with each partition serving as a test set once. The performance of each model validation was then averaged.

#### Identification of Optimal Time Window

In total, 6 weeks of data were collected for this study. As continuous and periodic data collection for long periods of time can be expensive and increase the risk of data loss, we investigated the minimum time window needed for reliable classification. First, we used an incrementally increasing time window to train the classification model, starting from day 1 and adding 1 day until we included all 42 days of data. We examined which time window would yield the highest overall accuracy, sensitivity, and specificity. We compared the performances of 3 classification algorithms (least absolute shrinkage and selection operator [LASSO]-penalized logistic regression, random forest, and support vector machine) to classify patients with FSHD and non-FSHD controls using the incremental time windows. Second, we used the optimal time window to train the classification model and evaluated how stable the classification performance would be for the remaining 5 weeks of data. Here, we evaluated the stability of the algorithm based on the generalization error of the trained classification model [23].

## Results

### Data Collected

In total, 58 participants (n=38, 66%, patients with FSHD and n=20, 34%, non-FSHD controls) participated in the study. We did not meet our goal of 40 patients because of difficulties in recruiting patients in an acceptable time span.

The female-to-male ratio was the same in both populations; however, the median age of the control participants without FSHD was lower than that of their counterparts with FSHD. Table 2 illustrates the demographic and disease characteristics of the participants enrolled in this study. The FSHD clinical scores and TUG scores remained relatively stable during the 6-week period (with a maximum intraparticipant change of 1 point for the FSHD score and 0.63 seconds for the TUG score).

**Table 2:** Demographics of patients with facioscapulohumeral dystrophy (FSHD) and controls without FSHD (N=58).

Demographics			Patients with FSHD		Non-FSHD controls
**Sex, n (%)**					
	Female	23 (61)		11 (55)	
	Male	15 (39)		9 (45)	
Age (years), mean (SD; range)			45 (14.5; 18-64)		33 (12; 23-69)
Weight (kg), mean (SD; range)			80 (16; 52-130)		78 (18; 56-129)
BMI (kg/m^2^), mean (SD; range)			26 (4; 20-44)		25 (5; 19-35)
FSHD clinical score, mean (SD; range)			5 (3; 1-13)		0 (0; 0-0)
Timed Up-and-Go test (seconds), mean (SD; range)			8.8 (35; 5-15.81)		7.8 (1.55; 6-12.09)

### Perceived Burden and Data Loss

**Figure 1:**
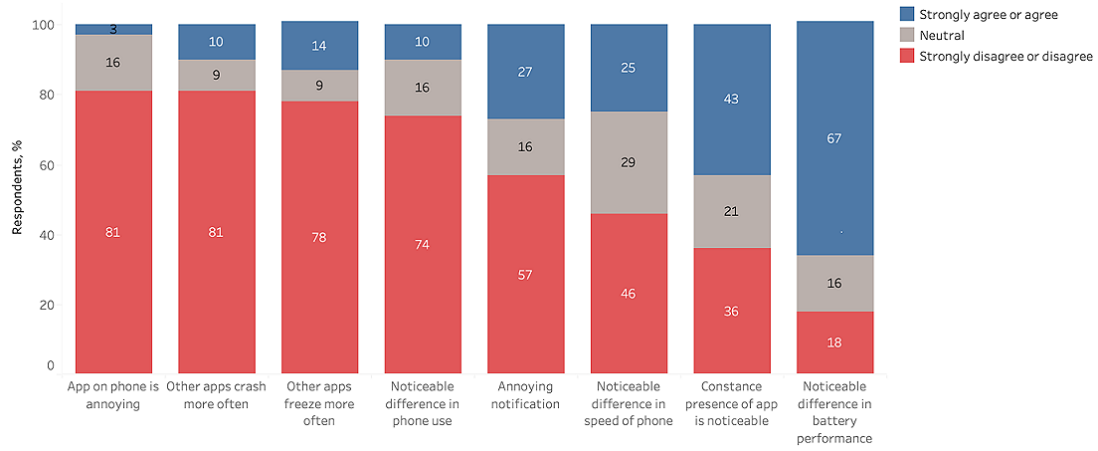
Feasibility and perceived burden of remote monitoring in patients with facioscapulohumeral dystrophy using smartphone-based technologies.

As shown in Figure 1, overall, 3% (2/58) of the participants found the app on their phone to be annoying. Furthermore, 67% (39/58) of the participants agreed that there was a noticeable difference in battery life, 43% (25/58) agreed that the constant presence of the app was noticeable on their smartphone, 28% (16/58) rated the constant visible notification as annoying, and 26% (15/58) of the participants noted a difference in the speed of their smartphone.

Data completeness is defined as having incoming data for each day of the clinical trial, except for the blood pressure and scale data, for which completeness is defined as having incoming data each week. As phone and SMS text messaging data are activity triggered and are aperiodic, it is not possible to know whether data were missing. Table 3 provides an overview of data completeness for the CHDR MORE app, Withings watch, Withings scale, and Withings blood pressure monitor and their respective sensors.

**Table 3:** Overview of data completeness. The data completeness shows what percentage of data was collected among the participants during the 42 days of the study; hence, in total, there should be 2436 daily instances and 232 weekly instances.

Sensor	Feature	Overall data completion			
		Patients with FSHD^a^		Controls without FSHD	
		n (%)	N	n (%)	N
Microphone (smartphone)	Voice activation	1181 (74)	1596	688 (81.9)	840
Accelerometer (smartphone)	Phone acceleration	1260 (78.95)	1596	656 (78)	840
Google Places (smartphone)	Places	1109 (69.49)	1596	616 (73.33)	840
GPS (smartphone)	Relative location	1373 (86.03)	1596	785 (93.45)	840
App use (smartphone)	Use event aggregate	1404 (87.97)	1596	779 (92.74)	840
Withings blood pressure monitor	Blood pressure and heart rate	1452 (91.15)	1596	630 (75)	840
Withings scale	Body composition	173 (75.88)	228	88 (73.33)	120
Withings scale	Weight	205 (89.91)	228	108 (90)	120
Withings watch	Activity duration	1505 (94.3)	1596	744 (88.57)	840
Withings watch	Heart rate	1181 (74)	1596	588 (70)	840
Withings watch	Step count	1491 (93.42)	1596	708 (84.29)	840
Withings watch	Sleep summary	1408 (88.22)	1596	685 (81.55)	840

^a^FSHD: facioscapulohumeral dystrophy.

### Feature Selection

Several features were manually excluded before modeling. Because of the number of participants missing body composition data, we excluded all the body composition data with the exception of weight. Furthermore, we excluded SMS text message use features and app categories that were only used by only 5% (3/58) of the participants.

### Identification of Optimal Time Window and Classification Performance

**Figure 2:**
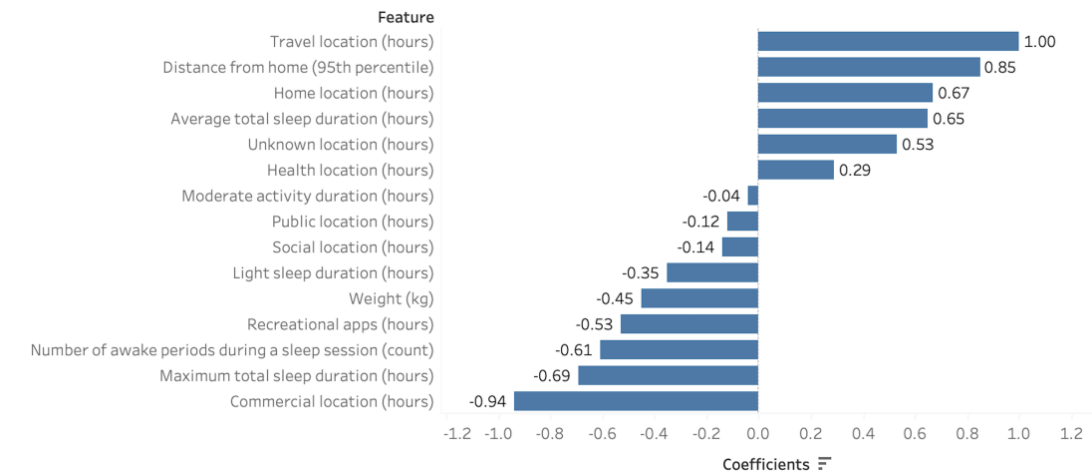
Selected features for classifying patients with facioscapulohumeral dystrophy and those without facioscapulohumeral dystrophy based on the composite data set using all 6 weeks of data and the least absolute shrinkage and selection operator–penalized logistic regression model. Unstandardized estimated coefficients indicate the direction of the association between the feature and the classification label.

Using all 6 weeks of data, the optimal classification model (LASSO-penalized logistic regression) achieved 93% accuracy, 100% sensitivity, 80% specificity, and 85% MCC. This classification model identified 15 features that were relevant for differentiating between patients with FSHD and non-FSHD controls. Specifically, features such as app use, weight, location, physical activity, and sleep were important for differentiating between the 2 populations (Figure 2). Table 4 shows the predictive features and their positive or negative associations with the classification label. The predictive features indicate that the participants in the group consisting of patients with FSHD were less likely to engage in moderate physical activity and spend time on recreational apps such as entertainment apps, music and audio apps, video players and editing apps, and games. The predictive features also showed that the participants in the group consisting of patients with FSHD were more likely to spend more time at home and health locations than their non-FSHD counterparts. Table 5 provides a summary of the number of selected features and the respective performance metric for each of the data sets fitted to the 6-week LASSO-penalized logistic regression model. The table illustrates that the composite data set model outperformed the models fitted to the social, physical activity, and biometric data sets. The MCC is used to select the best model because it corrects for class imbalances. The scores of the individual data sets are included to give an overview of their performance on their own. The MCC values of the social activity, physical activity, and biometric logistic regression models were 52%, 38%, and −21%, respectively.

**Figure 3:**
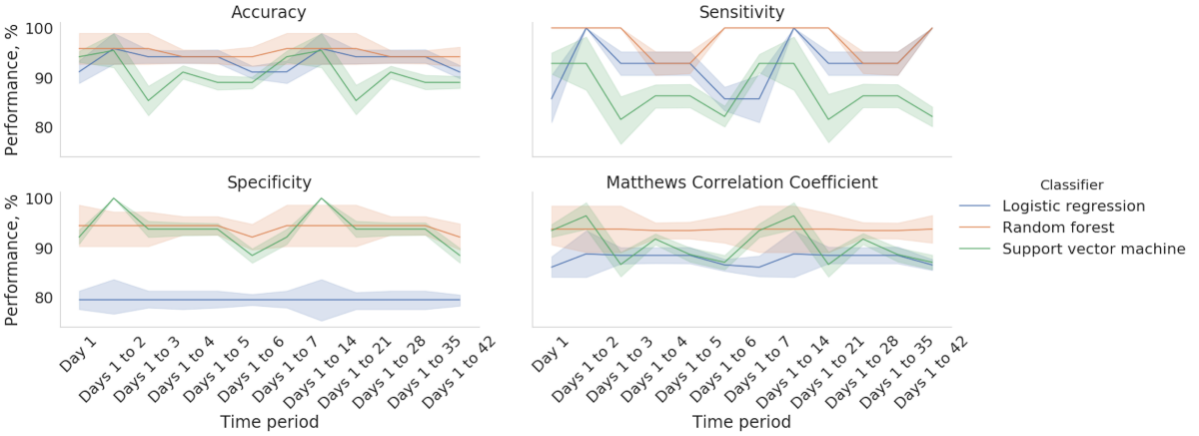
Performance of the incremental classification predictions for 3 classifiers (logistic regression, random forest, and support vector machine). The x-axis shows the time window for training the classification models starting from day 1 to day 42. The error bands represent the SD of the classification performance for the 5-fold cross-validation.

**Figure 4:**
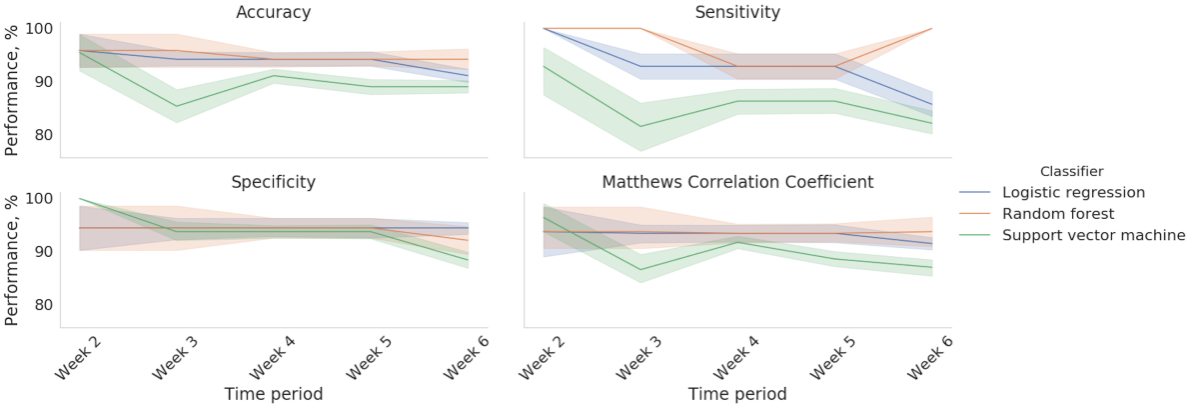
Performance of 3 classifiers (logistic regression, random forest, and support vector machine) trained on the week 1 data and used to predict the classification diagnosis of the subsequent weeks of data. The error bands represent the SD of the classification performance for the 5-fold cross-validation.

**Figure 5:**
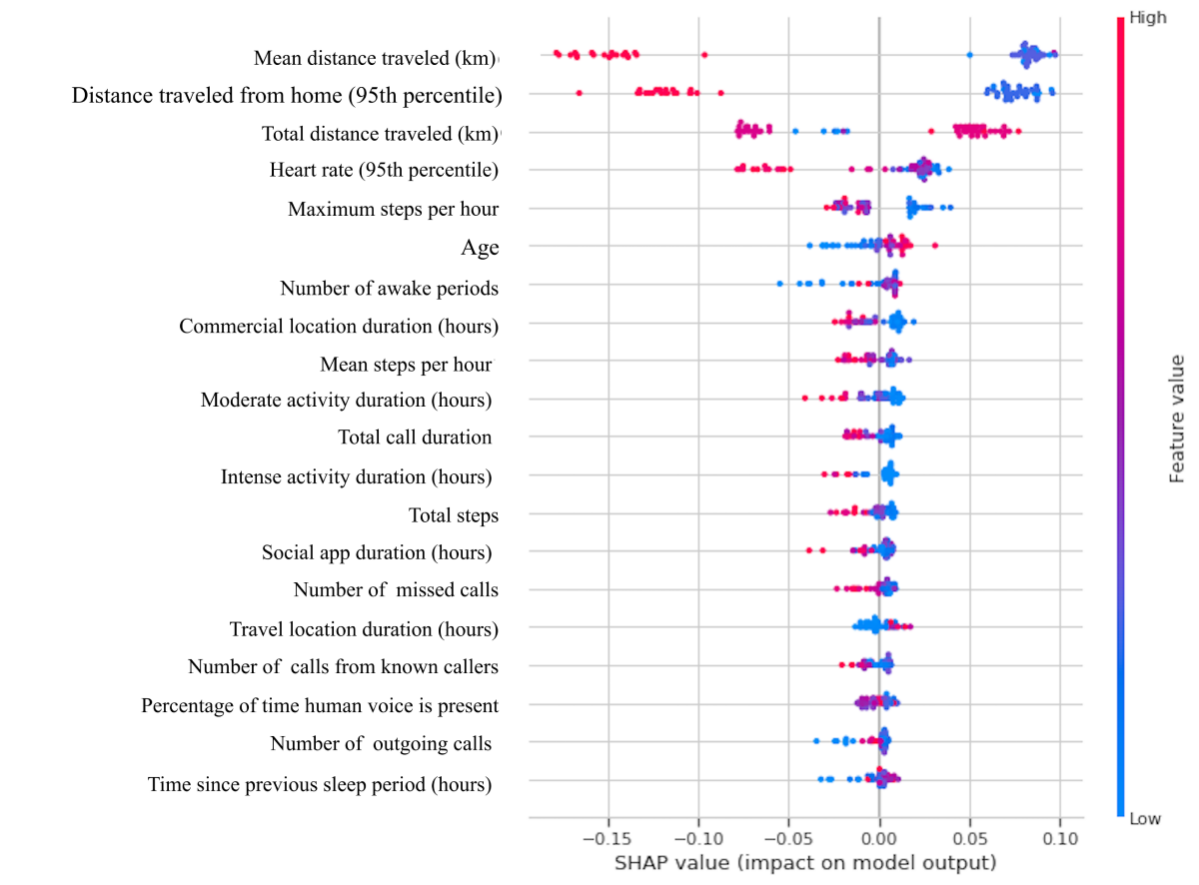
Shapley additive explanations (SHAP) summary plot based on a random forest classifier that was trained on the week 1 data. The x-axis shows the feature importance, where features are ranked in descending order. The y-axis shows the SHAP value that illustrates the direction of the association between the feature and facioscapulohumeral dystrophy severity. The color scheme reflects the probability of a participant being classified as a patient with facioscapulohumeral dystrophy.

As for identifying the optimal time window for accurately classifying the patients with FSHD and non-FSHD controls, we found that training the random forest on the data collected on the first day and the data collected during the first week yielded an accuracy, sensitivity, specificity, and MCC of 95.8%, 100%, 94.4%, and 93.8% (Figure 3). This approach outperformed the classification models that were trained on all 6 weeks of data. We also trained classification models on the first week’s data and fitted the data from subsequent weeks to assess the stability of the classification performance over time (Figure 4). We found that the random forest achieved the best overall performance, with a mean accuracy, sensitivity, specificity, and MCC of 95% (SD 0.9%), 97.6% (SD 3.6%), 94.1% (SD 0.9%), and 93.6% (SD 0.1%), respectively. Figure 5 provides a Shapley additive explanations plot that illustrates the magnitude and direction of the effect of a feature on a prediction. Of the 20 selected features, the top 5 (25%) most important features for the classification were mean kilometers traveled, 95% maximum distance from home, total kilometers traveled, 95% highest heart rate, and intense activity duration. For each of these features, the participants in the group consisting of patients with FSHD had lower scores than the non-FSHD controls.

**Table 4:** Selected features for classifying patients with facioscapulohumeral dystrophy and controls without facioscapulohumeral dystrophy based on the complete 6-week composite data set. Unstandardized estimated coefficients indicate the direction of the association between the feature and the classification label.

Feature category and feature			Unstandardized estimated coefficient
**Activity**			
	Moderate activity duration	−0.04	
**App**			
	Time spent on recreational apps	−0.53	
**Body**			
	Weight (kg)	−0.45	
**Location**			
	Distance from home: 95%	0.85	
**Time spent at location**			
	Travel location	1.00	
	Home location	0.67	
	Unknown location	0.53	
	Health location	0.29	
	Public location	−0.12	
	Social location	−0.14	
	Commercial location	−0.94	
**Sleep**			
	Average total sleep duration	0.65	
	Light sleep duration	−0.35	
	Number of awake periods during a sleep session	−0.61	
	Maximum total sleep duration	−0.69	

**Table 5:** Summary of number of selected features and the respective performance metric for each of the data sets used to classify the patients with facioscapulohumeral dystrophy from the controls without facioscapulohumeral dystrophy.

Data set	Number of selected features	Accuracy (%)	Sensitivity (%)	Specificity (%)	MCC^a^ (%)
Composite	15	93	100	80	85
Biometric	5	57	89	0	−21
Social	10	79	90	60	52
Physical activity	13	71	78	60	38

^a^MCC: Matthews correlation coefficient.

## Discussion

### Principal Findings

We investigated the feasibility of monitoring and characterizing the physical, social, and biometric features of patients with FSHD and non-FSHD controls using remote monitoring technologies. The use of the remote monitoring platform was well tolerated by all participants. Next, we found that a minimum of 1 day of data and a maximum of 1 week of data can be used to reliably classify the 2 populations. In fact, an FSHD classification model trained on data from a shorter time window outperformed a classification model trained on data from the entire 6-week period. Furthermore, we illustrated that a classification model trained on the first week’s data yielded stable and reliable classification predictions across the remaining 5-week period.

Most (37/58, 64%) of the participants tolerated the CHDR MORE app constantly running on their smartphone (Figure 1). Of the 58 participants, only 2 (3%) stated that the app was annoying. However, the results show that some of the participants agreed that there was a noticeable difference in smartphone speed performance (14/58, 25%), stability (8/58, 14%), and overall battery life (39/58, 67%). Therefore, the presence of the app was noticeable for some (25/58, 43%) of the participants. The decrease in smartphone performance (ie, speed, stability, and battery performance) was likely due to the continuous sampling of the sensors. As this was the first study in this specific patient group with this platform, all smartphone sensors were frequently sampled to capture all possible features. With the collected data in this study, we identified the features that are useful in differentiating between patients with FSHD and non-FSHD controls. In future studies, noncontributing raw data such as data from the accelerometer and gyroscope (both sampled at 5 Hz) can be turned off to reduce the burden on the battery performance and overall user experience. We do not know for certain whether, and how, the noticeability of the app affects participants’ behavior. Of the 58 participants, 6 (10%) stated that they noticed a change in smartphone use for themselves, which may mean that they changed their behavior. Therefore, participants will know that they are participating in a study and that they are being constantly monitored even if the app is perfectly optimized. As a result, some sort of change in behavior can be expected.

As for the user experience and perceived burden questionnaire, we designed a questionnaire based on our own experiences with smartphone use and the predicted effects of the CHDR MORE app on smartphones. This questionnaire was not validated in any other study. At the time of designing the study, there were no validated and published smartphone app questionnaires that would fit our purpose. For example, the mHealth App Usability Questionnaire [24] focuses more on *active* smartphone apps, where there is interaction between the app and the participants. The CHDR MORE app is a *passive* app, requiring almost no interaction between the app and the user. Therefore, the questions should be more focused on the indirect effects of the app, such as more frequent crashes in other apps, subjective loss of snappiness of the operating system, or issues with battery performance. Although our questionnaire is not validated, it was considered the best way to accurately capture the perceived impact of the CHDR MORE app on smartphone use.

Feature selection is one of the most important processes for building a classification model. The inclusion of irrelevant features can confound the interpretability of the model because potentially predictive features would be excluded and therefore seem to be irrelevant. For example, because the patients with FSHD had more text-related activity than the non-FSHD controls, the SMS text messaging features were selected as important classification features. Given that the SMS text messaging features were not deemed clinically relevant because only 55% (21/38) of the patients with FSHD and 50% (10/20) of the non-FSHD controls actively sent outgoing SMS text messages and the majority of the SMS text messages were exchanged with unknown contacts, we excluded the SMS text messages as a feature. As a result, features that were initially not selected by the model for inclusion, such as sleep, were now deemed important features. The SMS text messaging features masked the relevance of other potentially predictive features. The features that researchers manually choose to include or exclude will influence the interpretability and stability of the model. It should be noted that although SMS text messaging features were excluded, features regarding instant messaging app use were included.

Our classification models allowed for the identification of a stable set of features that were distinctive of FSHD symptomology. We believe that identifying which remotely monitored features are relevant to FSHD can be a first step toward continuous monitoring of symptom severity and disease progression. For example, our classification model identified sleep as a relevant feature for classifying patients with FSHD. Other studies have found that patients with FSHD typically experience sleep anomalies because of anxiety, respiratory muscle dysfunction, and pain [25-27]. This illustrates that the CHDR MORE platform is sensitive enough to detect and monitor sleep anomalies among individuals with FSHD outside of the clinic. Furthermore, location-related features were relevant for differentiating between the 2 populations. In this study, the patients with FSHD spent more time at home, in areas with public transportation, or at health locations than the healthy participants. Patients with FSHD face a range of physical challenges because of the functional deterioration in the affected muscular regions. Consequently, patients with FSHD may become more home bound and more reliant on public transportation for travel, as well as require more visits to their physicians. In conclusion, the CHDR MORE platform provides data that can be used to show differences in the daily lives of patients with FSHD and controls without FSHD.

We demonstrated that there is a trade-off among the classification accuracy, the number of sensor measurements, and the duration of the monitoring period. Previous studies have demonstrated that using data from multiple sensors improves the detection of mental and physical health status compared with using data from a single sensor [28-30]. We illustrated that social activity, physical activity, and biometric data alone are insufficient for the accurate classification of FSHD. Rather, the inclusion of data from the smartphone, smartwatch, and scale improves the performance of the FSHD classification algorithm. Although the modeling of multi-sensor data can be advantageous, it can lead to several practical limitations. The inclusion of more features can increase the model’s complexity and thus limit the model’s explainability. Furthermore, the inclusion of more sensors and a longer monitoring period can be more expensive, potentially limit the number of participants enrolled in a study, and increase the risk of data loss. Future studies will need to weigh the advantages and disadvantages of integrating smartphones, smartwatches, scales, and monitoring period into their remotely monitored FSHD clinical trials.

Despite the good performance of our model, this study includes some limitations. The patients with FSHD and non-FSHD controls were comparable except for the age demographic. The median age of the non-FSHD controls was approximately 13 years less than that of the patients with FSHD. Generally, the older the person, the less they tend to use their smartphone and, in particular, the less they tend to use communication and social apps [31]. When characterizing patients with FSHD and non-FSHD controls based on active smartphone use, the model may be biased because of the difference in age. However, as seen in the results, only 1 feature of active smartphone use—time spent on recreational apps—was included in the final model for the characterization of patients with FSHD, which may limit the impact of this difference. The other features used in the composite model consist of either physical activity features collected passively from the smartphone or biometric data collected from the Withings devices. Therefore, we believe that the impact of these *contaminated* data on the performance of our model is low.

The objective of our study was to capture continuous sensor data. However, these data can only be considered reliable when participants carry their smartphone and have it turned on all the time. During this study, all participants were instructed to do so. However, data captured when the participant was not carrying their smartphone could not be distinguished from data captured when the participant was carrying the smartphone. Therefore, all instances in which the smartphone is not carried or turned on result in unrepresentative data. These data get mixed in the *real* data because these moments cannot be filtered out of the data with full certainty, resulting in unreliable data. Of note, there is no easy solution to this problem. It would be difficult to continuously check whether the participants are carrying their smartphone using the built-in sensors. However, adherence to this requirement is an important aspect in remote data collection, emphasizing the need for clear instructions on this adherence aspect to participants during training sessions before study start.

The level of data loss from the Withings scale indicates that improvement is needed to gather reliable scale data (Table 3). Data loss occurred for both the patients with FSHD and the non-FSHD controls, indicating that the loss of data was unlikely related to any of the FSHD symptoms. Although clear instructions were given at the beginning of the study and all participants received a manual with the same instructions, we believe that the data loss was caused by improper use of the scale by the participants. The weight measurement consisted of two parts: (1) measurement of weight and (2) measurement of body composition. Weight was determined first, followed by a blinking notification on the display during the measurement of body composition. This might have given the impression to the participant that the measurement had been completed, causing them to interrupt the second part of the measurement, resulting in an incomplete measurement. For future studies, we recommend incorporating a live training at the beginning of the study on the correct use of the scale.

Efficient clinical testing of any FSHD intervention or of any drug targeted at improving function of patients with FSHD or delaying disease progression requires the availability of clinical biomarkers that ideally change relatively rapidly over time; correlate with, and allow for, prediction of progression of the existing clinical severity and functional scores; and allow for identification of fast progressors. Using data collected in a home setting might provide a more comprehensive picture of the evolution of a patient’s overall condition over time. This study is a first step in the development and validation process of using data collected by a specific remote monitoring platform for use in patients with FSHD. The features described in this paper may be useful in further evaluating the impact of the disease and monitoring disease progression in patients with FSHD in the future [13]. More extensive data from longitudinal studies are needed to further define how social, physical, and biometric data collected remotely can be used to monitor symptoms.

### Conclusions

To conclude, this study illustrates that the collection of smartphone data and wearable data is acceptable to patients with FSHD and non-FSHD controls and can be used to differentiate between the 2 populations. We showed that remotely monitored end points can capture behavioral differences between patients and controls. Further longitudinal studies are warranted to study the potential of using a remote monitoring system for detecting FSHD symptom severity and possible drug effects.

## Appendix

Multimedia Appendix 1 Perceived burden questionnaire.

Multimedia Appendix 2 Additional background information of investigated classifiers.

## Abbreviations

CHDR: Centre for Human Drug Research
CHDR MORE: Centre for Human Drug Research Monitoring Remotely
FSHD: facioscapulohumeral dystrophy
LASSO: least absolute shrinkage and selection operator
MCC: Matthews correlation coefficient
TUG: Timed Up-and-Go test

## Data Availability

The data that support the findings of this study are available from Facio Therapies, but restrictions apply to the availability of these data. However, data are available from the authors upon reasonable request and with permission of Facio Therapies.
