# Supplementary material for: Objective Monitoring of Facioscapulohumeral Dystrophy During Clinical Trials Using a Smartphone App and Wearables: Observational Study
Source: JMIR Form Res. 2022 Sep 13;6(9):e31775. doi: 10.2196/31775 (PMC9516375; doi:10.2196/31775)
Supplement: Multimedia Appendix 1 [file formative_v6i9e31775_app1.docx]

# Multimedia Appendix 1

**Perceived burden and user experience questionnaire**

Name:

Date:

| **Question** | **Answer** | | | | |
| --- | --- | --- | --- | --- | --- |
| There is a noticeable difference in how fast the phone is after installing the app. | *Strongly disagree*  *⃝* | Disagree  *⃝* | Neutral  *⃝* | Agree  *⃝* | Strongly agree  *⃝* |
| The constantly visible notification is distracting. | *Strongly disagree*  *⃝* | Disagree  *⃝* | Neutral  *⃝* | Agree  *⃝* | Strongly agree  *⃝* |
| There is a noticeable difference in battery life after installing the app. | *Strongly disagree*  *⃝* | Disagree  *⃝* | Neutral  *⃝* | Agree  *⃝* | Strongly agree  *⃝* |
| Other apps crash more often after installing the app (apps drop out and the Android home screen is seen again). | *Strongly disagree*  *⃝* | Disagree  *⃝* | Neutral  *⃝* | Agree  *⃝* | Strongly agree  *⃝* |
| Other apps freeze more often after installing the app (apps no longer respond to touching the screen). | *Strongly disagree*  *⃝* | Disagree  *⃝* | Neutral  *⃝* | Agree  *⃝* | Strongly agree  *⃝* |
| Do you notice that you use the phone differently after installing the app. (Not opening certain apps anymore, calling more or less often, messaging more or less often, etc.) | *Strongly disagree*  *⃝* | Disagree  *⃝* | Neutral  *⃝* | Agree  *⃝* | Strongly agree  *⃝* |
| The constant presence of the app is noticeable. | *Strongly disagree*  *⃝* | Disagree  *⃝* | Neutral  *⃝* | Agree  *⃝* | Strongly agree  *⃝* |
| The app on the phone is disruptive. | *Strongly disagree*  *⃝* | Disagree  *⃝* | Neutral  *⃝* | Agree  *⃝* | Strongly agree  *⃝* |

Comments:
