# Supplementary material for: Objective Monitoring of Facioscapulohumeral Dystrophy During Clinical Trials Using a Smartphone App and Wearables: Observational Study
Source: JMIR Form Res. 2022 Sep 13;6(9):e31775. doi: 10.2196/31775 (PMC9516375; doi:10.2196/31775)
Supplement: Multimedia Appendix 2 [file formative_v6i9e31775_app2.docx]

# Multimedia Appendix 2

**Additional background information of investigated classifiers**

*Logistic Regression*

Logistic regression is a supervised classification algorithm that models a binary dependent variable using a logistic function.[15] Logistic regression models the conditional probability of the response variable Y as a function of a variable X (*Pr(Y=1|X=x)*). The objective is to maximize the conditional logistic likelihood which is estimated with respect to the weight of the variables and bias parameters.[16] The final classification is dependent on the probability estimate with a threshold. To reduce the likelihood of overfitting, regularization can be used to reduce the degree of freedom. Lasso regularization penalizes the least important features and shrinks their respective coefficients to zero, therefore eliminating them from the regression model.[17]

*Random Forest Classifier*

Random Forests comprise of an ensemble of a multitude of decision trees. Each classification is the result of the mode of the classes’ outputs by averaging multiple deep decision trees, which are each trained on different parts of the same training data.[18] The two main advantages of Random Forest is that overfitting rarely occurs due to the tendency of eliminating decision trees and no a priori knowledge about the distribution is required.[19] However, the main disadvantage is that slight variation in the training data can result in vastly different decision tree structures.

Support Vector Machines

Support vector machines (SVMs) are supervised non-probabilistic binary classifiers.[20] Support vectors refer to the data points that lie closest to the decision boundaries (also known as the hyperplanes)[21] that define the two classes. SVMs aim to identify the optimal hyperplane that maximizes the margin between the two classes. SVMs are typically resilient to overfitting as the hyperplane is only dependent on a subset of the dataset rather than the relying on the entire dataset.[22]
